# Direct electrical control of IgG conformation and functional activity at surfaces

**DOI:** 10.1038/srep37779

**Published:** 2016-11-24

**Authors:** Paola Ghisellini, Marialuisa Caiazzo, Andrea Alessandrini, Roberto Eggenhöffner, Massimo Vassalli, Paolo Facci

**Affiliations:** 1 Department of Surgical Sciences and Integrated Diagnostics, University of Genova, Corso Europa 30, 16132 Genova, Italy; 2 Interuniversity Consortium INBB-Viale delle Medaglie d’Oro, 305, 00136 Roma, Italy; 3 Department of Physical, Informatic and Mathematical Sciences, University of Modena and Reggio Emilia, Via G. Campi 213/A, 41125 Modena, Italy; 4 CNR-Nanoscience Institute-S3, Via G. Campi 213/A, 41125 Modena, Italy; 5 CNR-IBF, Via De Marini, 6, 16149 Genova, Italy

## Abstract

We have devised a supramolecular edifice involving His-tagged protein A and antibodies to yield surface immobilized, uniformly oriented, IgG-type, antibody layers with F_ab_ fragments exposed off an electrode surface. We demonstrate here that we can affect the conformation of IgGs, likely pushing/pulling electrostatically F_ab_ fragments towards/from the electrode surface. A potential difference between electrode and solution acts on IgGs’ charged aminoacids modulating the accessibility of the specific recognition regions of F_ab_ fragments by antigens in solution. Consequently, antibody-antigen affinity is affected by the sign of the applied potential: a positive potential enables an effective capture of antigens; a negative one pulls the fragments towards the electrode, where steric hindrance caused by neighboring molecules largely hampers the capture of antigens. Different experimental techniques (electrochemical quartz crystal microbalance, electrochemical impedance spectroscopy, fluorescence confocal microscopy and electrochemical atomic force spectroscopy) were used to evaluate binding kinetics, surface coverage, effect of the applied electric field on IgGs, and role of charged residues on the phenomenon described. These findings expand the concept of electrical control of biological reactions and can be used to gate electrically specific recognition reactions with impact in biosensors, bioactuators, smart biodevices, nanomedicine, and fundamental studies related to chemical reaction kinetics.

The possibility of acting on biomolecules using an applied electric field is at the basis of many methods and approaches adopted in different contexts such as bioanalysis, diagnosis and therapy, nanobiotechnology, and molecular electronics^1^,^2^,^3^. This possibility stems from the fact that, in physiologic conditions, biomolecules possess net electric charges and generally have quite complex charge distributions^4^, which make them sensitive to the presence of external electric fields.

Just to quote some paradigmatic cases, electrophoresis is a clear example of the use of an electric field for distinguishing among biomolecules of different mobility (see, f.i., ref. 5). Electrophysiology measurements take advantage of the possibility of imposing a potential difference between the cytoplasmic part of a cell and the extracellular environment, gating the aperture of an ion pore^6^. In medicine, alternating and constant electric fields are exploited in some of the most advanced and widely spread diagnostic tools (e.g. X-ray tomography, MRI) and are also useful in therapy (e.g. ionophoresis, hyperthermia).

In the realm of nanotechnology, dielectrophoresis is used to provide fine positioning of molecular scale objects^7^,^8^, including biomolecules, in nanometer-scale gaps between electrodes. Furthermore, biomolecular electronics make use of biomolecules for technological tasks^9^ as components of electronic devices, sensors, etc., or exploit their intrinsic “electronic” functional activity for assembling bottom-up, single molecule devices^10^.

Moving forwards along the direction of exploiting electric fields to influence biomolecular behavior, an appealing possibility, although not much exploited in the literature, is that of manipulating complex biomolecules and bioreactions with submolecular precision.

The great potentiality of this approach emerges readily by considering the generally recognized relationship between conformation and function in biomolecules^4^. Indeed, biomolecular global functional states can be associated with sets of conformational ones and inter-conversion among different such sets could in principle modulate molecular function^11^.

Just a few attempts to implement a direct electrical control over biological reactions were reported so far to our knowledge. Particularly, we recall the action of transmembrane voltage on the functional state of voltage-gated ion channels and other membrane proteins^6^; the technological use of electric fields to attract/repel ssDNA molecules from electrode surfaces where they could hybridize with pre-immobilized probes^12^,^13^,^14^; the recent implementation of a bio-fuel cell taking direct advantage of the metabolism of a living being^15^. More specifically, the only report of a work aimed at controlling electrically immunological reactions for technological aims is that of Sivan and co-workers^16^. They used SPR to demonstrate the reversible stripping of specifically bound antibodies from an antigen-coated gold layer when a potential more negative than −0.5 V (*vs* Ag/AgCl) was applied to the gold substrate.

In the present work, we show that it is possible to implement an electrical drive to antibody-antigen reactions, relevant in immunosensing, based on a different principle, i.e., by taking advantage of: (i) proper surface bio-functionalization, and (ii) direct electrochemistry. With these two basic ingredients, we were able to drive electrically the conformation of surface-immobilized antibodies, thus achieving a modulation of their binding to the corresponding specific antigens. Key requirements involved (i) immobilizing IgG-type molecules with a unique orientation in such a way that specific recognition sites were exposed to the solution and, (ii) finding the conditions to affect IgG conformation with an electric field generated in solution by an electrically polarized gold substrate on which antibodies were immobilized.

Figure 1 anticipates the kind of molecular arrangement and operating principles for the direct electrochemical control of IgG conformation at an electrode surface.

To demonstrate our achievements, we have implemented an electrically tunable immunosensor and exploited several experimental techniques and approaches (electrochemical quartz crystal microbalance–ECQCM, electrochemical atomic force microscope-ECAFM, electrochemical impedance spectroscopy-EIS, electrochemically-assisted fluorescence microscopy) all providing complementary pieces of evidence converging to formulate the concept reported in what follows.

## Materials and Methods

Au substrates used in the various experimental techniques were invariably obtained by thermal evaporation of a 100 nm Au film on top of an adhesion-promoting 10 nm-thick Cr layer on mica, glass, or quartz supports. The evaporation parameters used were: base pressure 10^−6^ mbar, evaporation rate 0.2 nm/s. When used immediately after preparation, substrates were incubated without any other processing. In the case of delayed use, they were exposed to piranha solution (H_2_SO_4_/H_2_O_2_ 70/30 V/V) for 20 min, rinsed in abundant ddH_2_O and exposed to O_2_ plasma to clean them from atmospheric contaminants and carbon residues, immediately prior to ethanol rinsing (to reduce gold oxide) and use.

His-tagged protein A mutant was supplied by BioVision and used without further purification. It was reconstituted in H_2_O, according to the protocol suggested by the supplier, at a concentration of 1 mg/ml. Anti-goat antibodies and mouse anti-insulin were diluted in 150 mM PBS at a concentration of 100 ng/ml for incubation. Goat antibodies used as antigens were diluted at 100 ng/ml in 5 mM PBS.

Fitch-labeled insulin (Sigma) was diluted in 5 mM PBS at a concentration of 1 mg/ml.

### ECQCM

ECQCM was used to assess adsorption kinetics measurements of antigens in solution and the check the various biofunctionalization steps by measuring the changes in visco-eleastic load on the resonator surface as a function of incubation time and various reaction parameters.

AT-cut quartz crystals (ICM Co. Inc.) with a nominal resonance frequency of 10 MHz were used in all the experiments. A home-made flow chamber^17^ exposing just one quartz face to solution was connected to a PC-driven impedance analyzer (HP 4294A) measuring the whole resonance amplitude and phase spectra around 10 MHz, the fundamental resonator oscillation frequency, every 4 seconds. A Lorentzian curve was fit to the resonance one to increase the frequency resolution for the value corresponding to the curve-maximum. From these spectra, the variation in resonance frequency and the FWHM of the resonance frequency curve, proportional to dissipation, could be extracted.

The solution-exposed face of the QCM crystal was connected to an external potentiostat (Amel 2000) and served as a working electrode; a Pt and an Ag wires acted as counter, and quasi-reference electrode respectively (the use of the latter being dictated by the minute size of the measuring chamber).

The measuring chamber was fed by a syringe pump (Harvard Apparatus PHD 2000) used for injecting reagents.

### ECAFM

This technique was exploited to compare the molecular layer topography as a function of the applied potential.

A Bioscope I in stand-alone configuration equipped with a Nanoscope IIIA controller (Digital Instruments) was used in tapping-mode in liquid. Commercial cantilevers (Bruker, DNP) with a typical elastic constant of 0.24 N/m and resonance frequency of 8–10 kHz in liquid were used. Au substrates were connected to an external potentiostat (Amel 2000) that could drive their potential with respect to solution using electrodes placed inside the imaging cell. Measuring buffer consisted in 5 mM PBS, pH 5.5.

### EIS

Electrochemical impedance spectroscopy was used to check the characteristics (i.e. compactness) of the IgG layer as a function of the applied potential by measuring changes in effective layer capacitance. It was measured with an electrochemical workstation (Mod. 670 CH Instruments) in PBS 5 mM, pH 5.5 and 9.5, at a base potential of +/−100 mV *vs* Ag/AgCl reference electrode in the frequency range 0.01 Hz ÷ 100 kHz. Data were fitted with the equivalent circuit shown in Supplementary Figure S1.

### Fluorescence Microscopy

A laser scanning confocal microscope (Leica TCS SP2) was used for imaging biofunctionalized Au pads after incubation with fluorescently labeled (FITC) insulin. The microscope was equipped with FITC filters.

Samples were incubated off-line, using a bipotentiostat (PicoStat, Molecular Imaging) to drive the potential of the two separately addressable Au pads at +/−100 mV potential, respectively.

## Results and Discussion

The first step was that of developing a surface immobilization chemistry for antibodies on a gold electrode surface that enabled their preferential orientation, hence that of their F_ab_ fragments, towards the solution covering the electrode.

**Figure 1:**
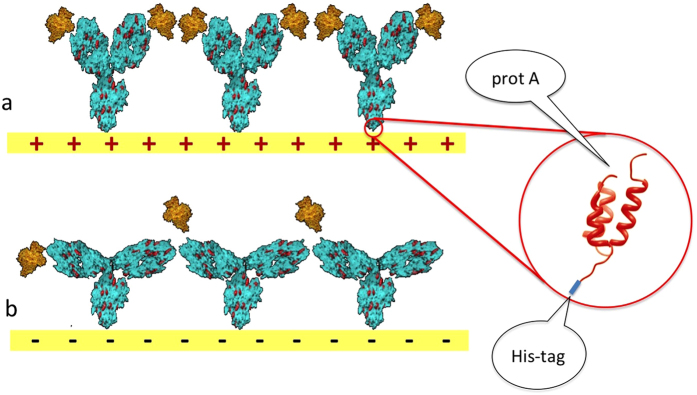
Representation of molecular arrangement and effect of substrate potential on the conformation and antigen binding ability of IgGs immobilized on Au electrodes. (**a**) A positive substrate potential repels IgGs’ positive charges (red dots) from the surface, pushing F_ab_ fragments towards the solution bulk; antigen binding from solution is thus favored. (**b**) A negative substrate potential attracts IgGs’ positively charged residues, pulling F_ab_ fragments towards the electrode surface; in this case, antigen binding is made less probable due to a decreased accessibility of specific recognition sites by antigens in solution. A uniform orientation of IgGs on the surface is achieved by the use of a His-tagged protein A layer, shown in the zoomed inset only, for the sake of clarity.

We used protein A from *Staphylococcus aureus*, a molecule expressed in the outer membrane of the bacterium, with a high binding affinity for F_c_ fragments. The molecule we used was a mutant featuring 5 F_c_-specific binding sites, deletions of non-specific adsorption sites, and most importantly for us, a 6xHis tag at its N terminus. Given its high affinity for gold^17^,^18^,^19^ and other metals^20^, the 6xHis tag was used for adsorbing a (sub)monolayer of protein A onto gold, providing unambiguous molecular orientation. The formed monolayer was then incubated with IgGs, giving rise to an IgG (sub)monolayer characterized by a preferential orientation, namely F_ab_ fragments effectively exposed towards solution (see Fig. 1). Such results were initially evaluated in an indirect way by measuring protein coverage and antigen binding ability of IgG monolayers immobilized by the technique above *vs* a more standard, non-orienting surface functionalization strategy, see Supplementary Figure S2.

For an electric field to act on the immobilized IgGs, the antibodies have to be electrically charged. Generally speaking, proteins at physiological pH possess a net positive charge brought about mainly by their accessible Lys, Arg and His residues^4^. This is also the case for IgGs in physiological conditions, whose isoelectric point is typically between 6 and 9^21^. If one shifts pH to values larger than 9, amines of alkaline residues are no longer protonated and the macromolecule is no longer charged. Conversely, operating around pH 6, one gets protonated exposed amines that can respond to an electric field.

The possibility for an immobilized IgG to respond to an external electric field with a conformation change is, moreover, due to the presence of two flexible hinges that connect F_ab_ fragments to F_c_. Such hinges, impart a remarkable flexibility to the protein^22^ that is also correlated with its antigen binding activity^23^.

Our idea was trying to influence IgG’s conformation by direct electrochemistry, i.e. by changing the potential of the substrate hosting IgGs with respect to the solution with the help of an external potentiostat. By adjusting the ionic strength of the solution to a proper value (typically 5 mM) it is possible to achieve a diffuse layer thickness of the order of the thickness of our molecular layer (10 nm, for a 1:1 electrolyte)^24^, enabling thus the action of the electric field on the antibody. Higher electrolyte concentrations limit the diffuse layer spatial extent severely (more than 2 orders of magnitude decrease by rising ionic strength from 10^−3^ to 1 M)^24^,^25^ and result useless for the proposed application (data not shown), where large (IgG size ≈ 10 nm) macromolecules are involved.

**Figure 2:**
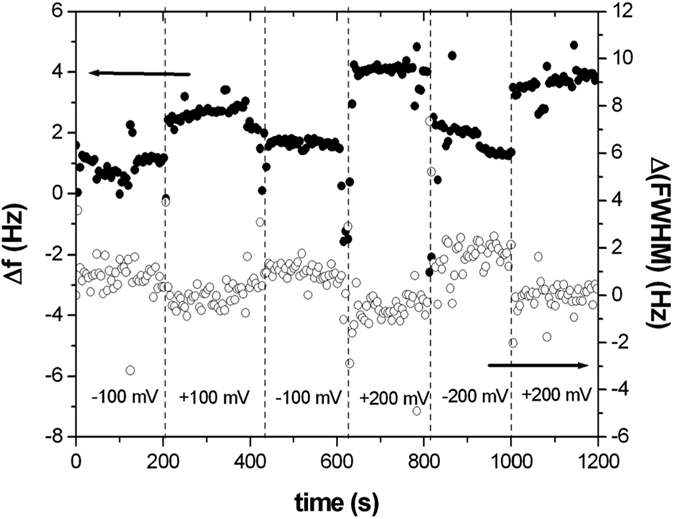
Response (resonance frequency variation, Δf, and resonance frequency distribution full width at half maximum variation, Δ(FWHM)) of prot A+ IgG layer to changes in substrate potential.

Once we had assembled a protein A-IgG monolayer, we performed electrochemical QCM measurements to investigate the dependence of its resonance frequency and dissipation upon changes in electrode potential. Having immobilized a layer of molecules on one electrode of a QCM, we measured its response to an electrical potential changing in sign and intensity (±100 ÷ ± 200 mV) inside a liquid cell equipped with a counter and reference electrode. Figure 2 reports the corresponding data. Both variations in resonance frequency and in FWHM (this last parameter being proportional to energy dissipation of the oscillator, i.e. to the compactness of the layer facing the QCM electrode) displayed a similar, although opposite in direction, stepwise behavior upon changing electrode potential. These data suggest qualitatively that the layer is responsive to those changes. One can think of potential-induced conformational changes increasing the apparent visco-elastic load measured by QCM since positive charges, hence molecular domains, are attracted towards the electrode surface when it is negatively biased. The conformation of the molecules on the surface can also affect the amount of trapped water which is moving together with the molecular layer affecting, on its turn, the resonance frequency of the oscillating crystal.

**Figure 3:**
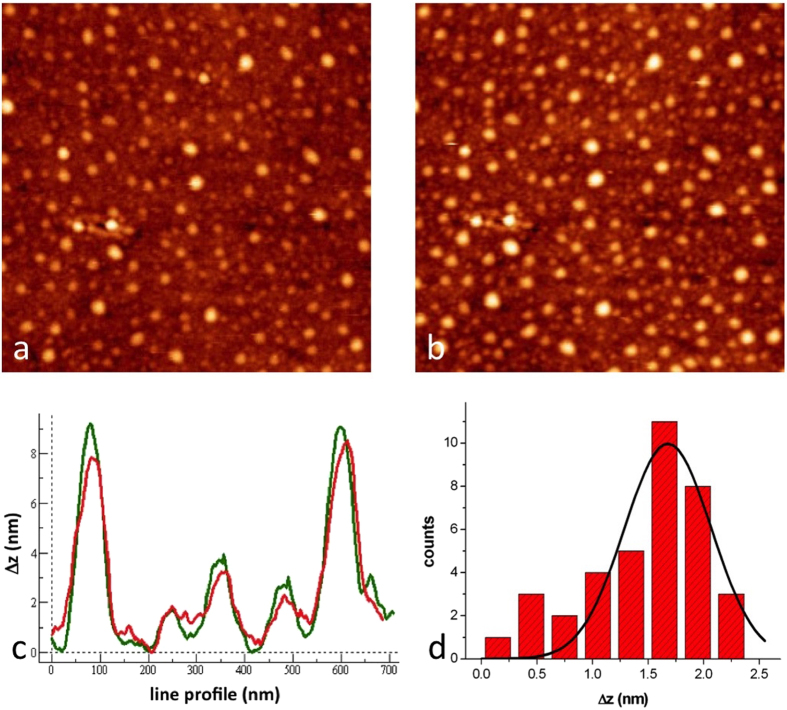
ECAFM results on the supramolecular edifice prot A+ IgGs as a function of substrate potential. (**a,b**) Topographic images measured at −100 mV (**a**), and +100 mV (**b**) *vs* SCE, respectively. (**c**) A representative pair of line profiles belonging to the two images. (**d**) The height difference histogram resulting from line profile analysis on the visible bumps.

Second, we characterized also at a microscopic level the changes induced by switching the sign of potential applied to a gold substrate on which the antibody layer was assembled. For doing that we used ECAFM, measuring topography on a single region during two consecutive scans performed at +100 and −100 mV, respectively while keeping the imaging force at the lowest possible value for both scans. Figure 3(a,b) reports two of those scans taken on the same area, showing a marked difference in height in the visible spots, associated to single IgGs. The analysis, performed by measuring corresponding line profiles (c) and building the resulting height difference histogram (d) reveals a difference in the average height of 1.7 nm between the case at +100 mV (F_ab_ fragment repulsion) and that at −100 mV (attraction). These results are consistent with a change of molecular conformation with substrate potential, likely connected with the rise of F_ab_ arms at positive, repulsive potential, and with lowering of them in the negative, attractive case. It is however fair to argue that the reported data do not figure out other possible conformational variations, such as a different molecular tilting induced by changes in substrate potential.

**Figure 4:**
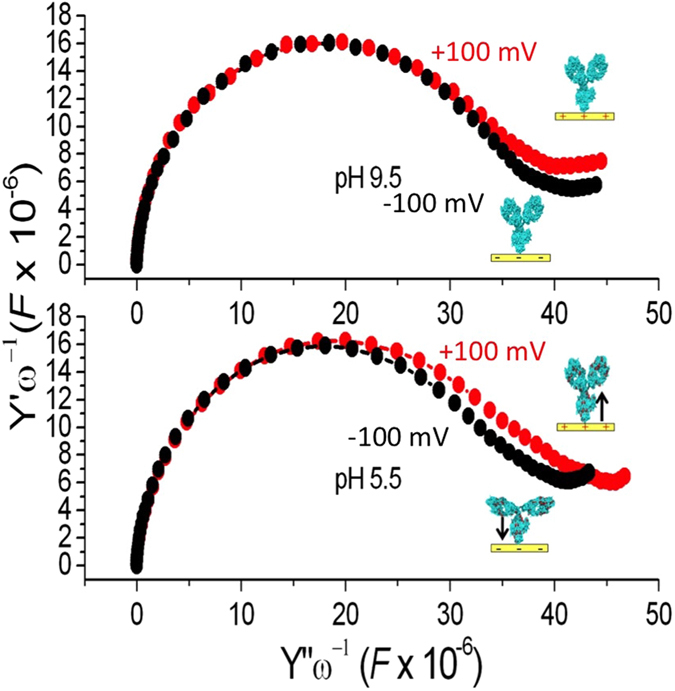
EIS of the supramolecular edifice prot A+ IgGs as a function of pH (5.5 and 9.5) and for two different base potentials (±100 mV). Data are shown in the complex admittance plane and report a different capacitance value of the construct measured at pH 5.5 (lower panel) for −100 mV (4.15 0.10^−7^ *F*) and +100 mV (4.67 0.10^−7^*F*).

Third, we measured EIS under different pH conditions corresponding to protonated (pH 5.5) and non-protonated (pH 9.5) amines, performing frequency sweeps on a different potential base (+/−100 mV). Figure 4 reports the corresponding data in the complex admittance plane. Whereas at pH 9.5 the two EIS traces measured at different base potentials are practically identical, when we switched to pH 5.5 a clear difference appeared in the behavior. Particularly, equivalent circuit analysis was performed with a simple net including a resistor and a constant phase element in series, see Supplementary Figure S1. The role of this element is that of accounting for an imperfect capacitor, as described by the *α* exponent, with 0 ≤ *α* ≤ 1 (*α* = 0 meaning an ideal resistor, while *α* = 1 an ideal capacitor). This simple model captured well the main features of the reported data showing a difference in capacitance in the case of +100 mV *vs* –100 mV pH 5.5 and an overall *α* value of 0.9, ascribable to the typical roughness of polycrystalline gold surfaces, see Supplementary Figure S1.

At −100 mV base potential, the equivalent circuit parameters show a very close resemblance to the case at pH 9.5. These results point to an effectively thicker/more compact film at the electrode/electrolyte interface in case of +100 mV base potential, i.e. when positively charged residues are repelled off the surface.

All these data concur to an overall picture for the protein A–IgG layer which is structurally sensitive to changes in sign and, partially, intensity of the potential of the electrode onto which the layer is grafted. Figure 1 summarizes a plausible mechanistic view of this phenomenon.

To deepen our understanding of the properties of these artificial supramolecular constructs, we addressed their functional behavior. Functionality, in the case of surface immobilized antibodies, refers to their ability to bind specific antigens. Particularly, our aim was that of evaluating the role of the various base potentials (conformations) on the antigen binding activity of the assembled antibody layers.

Towards that aim, we performed antigen adsorption kinetic measurements as a function of different parameters by means of ECQCM.

**Figure 5:**
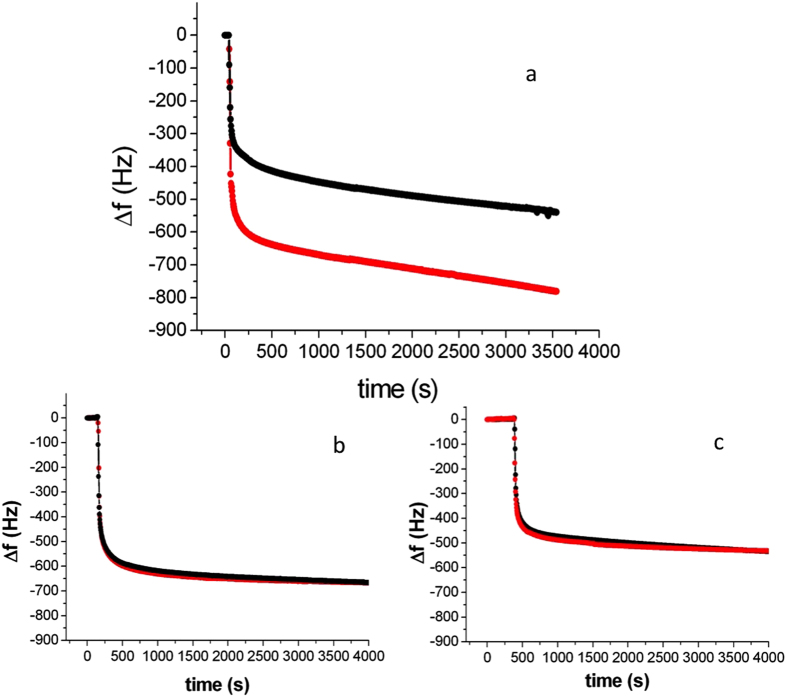
Adsorption kinetics measurements at different substrate potential values (red: +100 mV; black: −100 mV) and in different conditions; (**a**) pH 5.5; (**b**) pH 9.5; (**c**) pH 5.5, random molecular orientation. Antigen concentration was 0.1 mg/ml in 5 mM PBS.

Figure 5a shows a comparison of the adsorption curves (Δf) for two different base potentials (+/−100 mV). Antigen concentration was 0.1 mg/ml in 5 mM PBS. Whereas the kinetic features of the two curves are pretty much the same (double exponential decay with typical decay times of (70 ± 3) s and (1650 ± 100) s, respectively), the saturation levels appear to differ drastically, featuring a 65% higher value in case of positive potential. This result is crucial since it figures out any possible role of the direct electrostatic interaction between substrate and diffusing antigens (that, being positively charged in the chosen experimental conditions, should be repelled rather than attracted towards the surface by a positive potential and vice versa for a negative one) in increasing the saturation level. Rather, it is consistent with a picture that describes the increased binding ability of the layer at positive potential as due to a more effective exposure of the specific recognition sites located at the extremities of F_ab_ fragments. Conversely, it suggests that the decrease in binding ability registered at a negative potential is likely due to a more limited exposure of these molecular regions, possibly connected to an enhanced steric hindrance experienced by IgGs when their positively charged F_ab_ fragments are pulled towards the substrate by the action of a negative potential.

In order to further assess such results, we repeated the adsorption kinetics measurements in different pH conditions. Performing measurements at pH 9.5, i.e. where exposed amines are deprotonated and IgGs are virtually non-responsive to changes in potential, the results show no difference as a function of the substrate potential. Figure 5b reports the corresponding data. They point to a role of the charge state of immobilized IgGs in enabling conformational modifications, hence modulating binding activity.

A critical aspect for the overall picture to be satisfactory and convincing is that molecular orientation has to play a role. Indeed, only uniformly oriented IgGs can change coherently their orientation in response to a change in the sign of the substrate potential. Therefore, an assessment of the role of molecular orientation at surface appears to be fundamental. Towards that aim, we performed further antigen binding kinetics measurements using a different chemistry for surface immobilization of antibodies. By binding protein A to the electrode surface via its surface amines (brought about by Lys and Arg residues bound to the amine moiety of 2-mercaptoethilamine that was attached to the gold surface by its SH group and to protein primary amines via a glutaraldehyde linker), one no longer gets an uniformly oriented layer, rather a randomly oriented one due to the number of available binding sites all around protein surface (6 Lys and 1 Arg, see f.i. PDB entry 2JWD). As a result, also the IgGs captured by the protein A layer will be non-uniformly oriented. Supplementary Figure S2, left panel, reveals that IgG coverage in the case of oriented construct exceeds by 20% that of randomly oriented one.

Measuring antigen binding kinetics at ±100 mV substrate potential in this case, results in the curves reported in Fig. 5c. As it is evident, no effect of potential is detectable, since the two curves are substantially identical. This further evidence is consistent with the concept of an IgG layer whose uniform orientation, with F_ab_ fragments facing the bulk of the solution, achieved as a result of protein A uniform orientation, enables the activity modulation effect of an electric field as that arising from the IgG-supporting metal substrate.

**Figure 6:**
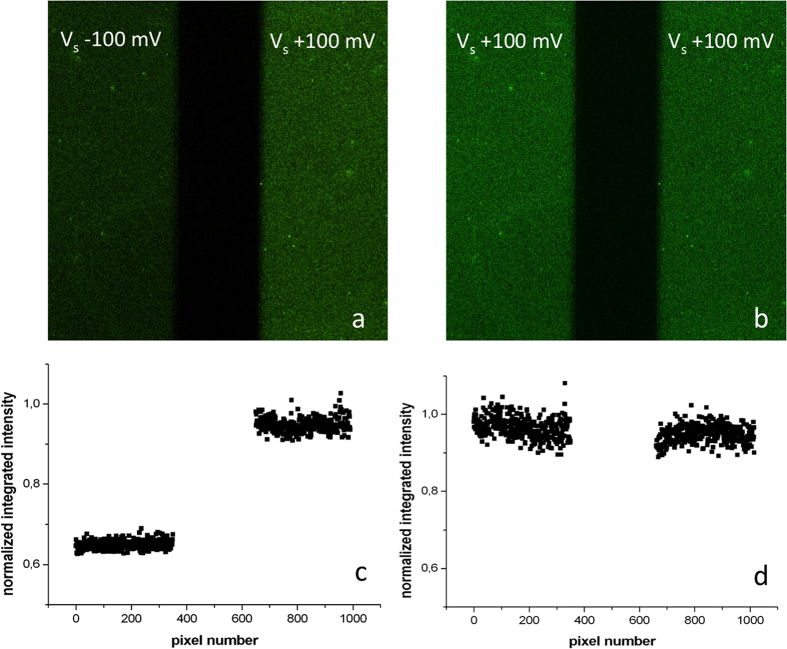
Confocal fluorescence imaging of two separately addressable gold pads biofunctionalized with prot A+ IgG and exposed to a solution of FITC-labelled insulin while kept at different substrate potentials. Image size 100 × 100 μm^2^. (**a,c**) pH 5.5; (**b,d**) pH 9.5. Note the lower intensity of the left pad in (**a**) (−100 mV). Panels c and d report the normalized integrated fluorescence intensity along columns for the two pads of images a and b.

The role of the applied electric potential has also been confirmed by performing antigen-binding measurements by means of a different technique, i.e. electrochemically assisted fluorescence microscopy. Two separately addressable gold pads were functionalized with uniformly oriented IgG layers, driven at two different potentials (±100 mV) while incubated with a fluorescently labeled specific antigen (insulin). In a control measurement, both electrodes were kept at +100 mV. After 1 h incubation, the sample was rinsed in buffer and imaged. Figure 6 reports the corresponding fluorescence images along with the computed normalized fluorescence intensity calculated by summing over the rows the collected light intensity in the two images. Once more (Fig. 6a), the role of the positive potential was that of eliciting a higher amount of antigens bound to the antibody layer in the case of incubation at a positive potential (confirming data shown in Fig. 5a).

It is plausible that the different behavior of the IgG layer subjected to electric fields of different sign could be traced back to the different molecular conformation, hence to the different exposure that F_ab_ fragments achieve in the two different configurations. A positive substrate potential tends to push F_ab_s off the substrate towards the solution, enabling a more efficient antigen capture due to a more efficient F_ab_ exposure. Conversely, a negative substrate potential, pulling F_ab_s towards the substrate, could decrease their exposure to the solution, hence to antigens, by enhancing the steric hindrance experienced by those fragments crowded on the surface, thus resulting in a smaller amount of captured antigens.

The electrostatic nature of the reported behavior is confirmed both by the described role of solution pH and by that of ionic strength, whose increase washes out any detectable effects (data not shown) on the molecular construct, consistently with the corresponding decrease of the diffuse layer thickness and related lack of effects of the substrate electric field on the charged moieties of the antibody layer.

It is straightforward to argue that reported results could find appealing application in immunosensor/bioactuator technology, as well as in the implementation of smart/programmable biosurfaces in nanomedicine. The described approach to the electrostatic control of molecular motion is also potentially significant for fundamental studies on chemical reaction kinetics. Other applications envisage the control of biocatalytic activity in enzyme biochips as well as the modulation of receptor-ligand interaction.

Finally, we believe that the described technology can pave the way to the future electrical control of more complex biological reactions and phenomena, even at cellular level, f.i. modulation of the activity of excitatory neurons as related to the redox-dependent functionality of GABA_A_ receptors, and that of gene expression level in suitable bacterial pathways shown to be redox dependent^26^.

## Additional Information

**How to cite this article**: Ghisellini, P. *et al*. Direct electrical control of IgG conformation and functional activity at surfaces. *Sci. Rep.* **6**, 37779; doi: 10.1038/srep37779 (2016).

**Publisher’s note:** Springer Nature remains neutral with regard to jurisdictional claims in published maps and institutional affiliations.

## Supplementary Material

Supplementary Information
