# Supplementary material for: Direct electrical control of IgG conformation and functional activity at surfaces
Source: Sci Rep. 2016 Nov 24;6:37779. doi: 10.1038/srep37779 (PMC5121884; doi:10.1038/srep37779)
Supplement: Supplementary Information [file srep37779-s1.pdf]

Supplementary Information to:

Direct electrical control of IgG conformation and functional activity at surfaces

Paola Ghisellini[1], Marialuisa Caiazzo[2,3], Andrea Alessandrini[2,3], Roberto Eggenhöffner[1], Massimo Vassalli[4], Paolo Facci[4*]

[1]Department of Surgical Sciences and Integrated Diagnostics, University of Genova, Corso Europa 30, 16132 Genova, Italy; & Interuniversity Consortium INBB - Viale delle Medaglie d'Oro, 305, 00136 Roma, Italy.

[2]Department of Physical, Informatic and Mathematical Sciences, University of Modena and Reggio Emilia, Via G. Campi 213/A, 41125 Modena, Italy.

[3]CNR-Nanoscience Institute-S3, Via G. Campi 213/A, 41125 Modena, Italy.

[4]CNR-IBF, Via De Marini, 6, 16149 Genova, Italy.

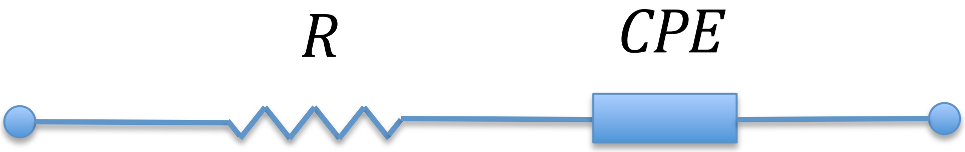

$$Z_{tot} = R + Z_{CPE} = R + \frac{1}{C(j\omega)^{\alpha}}; \quad 0 \leq \alpha \leq 1$$

S1. Equivalent circuit and corresponding total impedance used to model EIS measurements. R accounts for solution resistance, CPE (Constant Phase Element), account for the imperfect nature of the double layer capacitance. The value of α accounts for electrode surface roughness (α = ½ for an ideally porous electrode and α = 1 for a perfectly smooth one)[1].

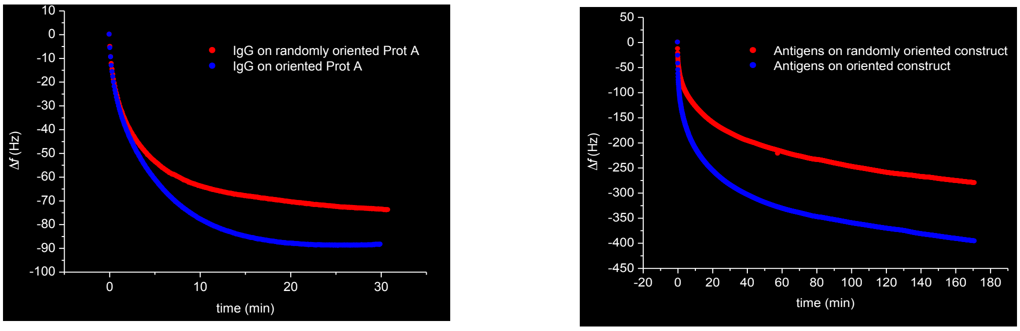

S2. Left panel reports the adsorption kinetic measurements of anti-goat IgGs on a preformed layer of protein A chemisorbed on gold by two different strategies: blue dots – by 6xHis tag at protein A N-terminus; red dots – by protein A surface amines (Lys and Arg). In particular, gold surface was incubated with 1 mg/ml mercaptoethylamine followed by 5% glutaraldheyde water solution. These two different functionalization strategies yield respectively a preferentially oriented protein A layer and a randomly oriented one. Both the constructs were then exposed to IgGs. The oriented construct allowed for a 20% higher IgG coverage.
Right panel reports the corresponding adsorption kinetics when antigens were exposed to the two aforementioned constructs. Here the “oriented” construct (blue dots) features a 30% higher coverage than the randomly oriented one (red dots), confirming the effect of the protein A orienting layer in both improving surface coverage and IgGs’ binding ability due to optimal antibody orientation.

References

1. Whitehouse, C. O’Flanagan, R. Lindholm-Sethson, B. Movaghar, B. Nelson, A. Application of Electrochemical Impedance Spectroscopy to the Study of Dioleoyl Phosphatidylcholine Monolayers on Mercury. *Langmuir*, **20**, 136-144 (2004).
